# Fabrication of reinforced and toughened PC/PMMA composites by tuning the migration and selective location of graphenes during melt blending

**DOI:** 10.1039/d0ra04790b

**Published:** 2020-08-03

**Authors:** Shigui Peng, Min He, Zhao Yang, Kai Zhang, Bin Xue, Shuhao Qin, Jie Yu, Guomin Xu

**Affiliations:** Department of Polymer Material and Engineering, College of Materials and Metallurgy, Guizhou University Guiyang 550025 China 410034801@qq.com; National Engineering Research Center for Compounding and Modification of Polymer Materials Guiyang 550014 China

## Abstract

In this work, we creatively obtained reinforced and toughened PC/PMMA/GNs composites by tuning the migration and selective location of graphene nanosheets (GNs) during melt blending. TEM results revealed that the migration of GNs in PC/PMMA blends during melt blending always existed no matter how the GNs were introduced, and most of them exclusively localized at the interface of PC and PMMA phase due to interfacial effects. The migration of GNs could refine the size of the dispersed phase, and the exclusive localization of GNs at the interface show obvious interfacial compatibilizing effects, leading to improved mechanical properties of the composites. It was found that the composite prepared by one-step compounding showed significant enhanced strength and toughness with addition of mere 0.05 wt% GNs and the tensile strength and elongation of the composite increased by about 62.96% and 94.54%, respectively as compared to the PC/PMMA blends. Moreover, the composite prepared by one-step compounding also showed improved thermal conductivity at the same time, indicating excellent comprehensive properties. It is believed that tuning the migration and selective localization of GNs open perspectives for the development of high-performance polymer composites.

## Abstract 2

By adjusting the dispersion and distribution of graphene nanosheets (GNs) filler in polycarbonate/polymethyl methacrylate (PC/PMMA) composites, only 0.05 wt% GNs filler can significantly improve the strength and toughness of the composites.

## Graphical abstract

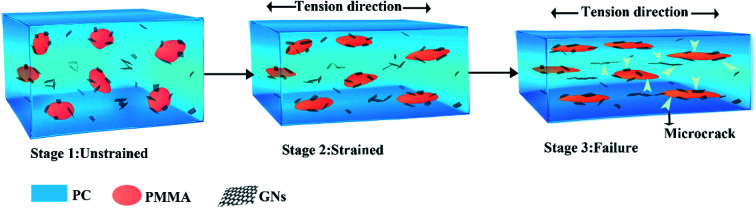

## 1 Introduction

The migration and selective location of layered nanoparticles in polymer blends during melt blending have been widely studied in recent two decades as they offer opportunities to obtain materials with excellent properties.^1–6^ In general, layered nanoparticles could selectively distribute in dispersed phase, matrix phase or an interface of polymer blends under proper thermodynamic and kinetic factors;^2^ this selective distribution determine the ultimate properties of blends by affecting the microstructure. If the distribution of layered nanoparticles can be tuned in a controlled manner, a polymer blend with tailored performance can be prepared.^6,7^ In fact, the controlled preparation of locally distributed layered nanoparticle/polymer composites with tailored properties is always one of the ambitions that researchers focus on.

Graphene nanosheets (GNs) have been one of the most widely concerned layered nanoparticles in recent years due to their excellent electrical, thermal, mechanical properties and large aspect ratios.^8–10^ Similarly, GNs also show migration and selective location during melting process, and this selective distribution shows significant effect on the final properties of the blend. Sharif *et al.* showed that PE/HIPS/GO composites exhibit remarkably enhanced strength because GO nanosheets localize at the interface of the blend and minimize the interfacial tension of PE and HIPS.^11^ Zhu *et al.* also found that the thermal conductivity of polymer blends could be greatly enhanced when GNs are trapped at the interface of the blend, and the blend achieved an extremely low thermal percolation threshold.^5,12^ Differently, Ray *et al.*^3^ reported that enhanced mechanical and thermal properties of biodegradable polylactide/poly(ε-caprolactone) (PLA/PCL) blends could also be achieved by selectively distributing functionalized GNs in the dispersion PCL phase, as the functionalized GNs improve the crystallization property of PCL. Obviously, still there is no consistent cognition regarding the effect of selective distribution of GNs on the performance of the polymer blend. In fact, the migration and selective location of layered nanoparticles during the melting process and its effect on microstructure and final property of the polymer blend are very complicated, dependent on the specific thermodynamic and kinetic conditions such as components, surface property of the nanoparticles, melt-compounding sequences and mixing times of the polymer blend, and shear rates.^4,11,12^ Despite the above-mentioned attempts, the migration mechanism and effect of selective location of GNs on the property of polymer blends are still insufficient, which need to be supplemented and summarized. Therefore, numerous further specific studies are still needed.

In addition, it is known from the above analysis that most of the current studies focus on the effect of selective location of GNs on electrical and thermal properties, and few research studies report the fabrication of reinforced and toughened polymer blends by selective location of GNs. In this study, we amazingly found that a reinforced and toughened polycarbonate/polymethyl methacrylate (PC/PMMA) blends could be prepared by selectively localizing GNs in the interface of the blends. The PC/PMMA blends was taken as the research object because the blend is of interest in the field of 5 G communication equipment due to good thermal stability, scratch resistance and toughness. Three different blending sequences were employed to tune the migration and distribution of GNs, and our effort focuses on studying the effect of distribution of GNs on their mechanical and heat-conducting properties and the relevant mechanism. It is believed that this study should provide a method for fabricating a tailored mechanical and heat-conducting performance polymer blend by tuning the selective location of layered nanoparticles on an industrial scale.

## 2 Experimental materials and procedures

### 2.1 Materials

PC (PC-122) with a density of 1.2 g cm^−3^ and a melt flow rate of 22 g/10 min was purchased from Taiwan Qimei Chemical Co. Ltd. PMMA (PMMA-v040) with a density of 1.19 g cm^−3^ and a melt flow rate of 16 g/10 min was purchased from French Akoma Chemical Co. Ltd. GNs with a diameter of 1 μm^−3^ and a sheet number of 3–5 layers were purchased from Qingdao Huagaomolene Technology Co. Ltd.

### 2.2 Sample preparation

Before the melting process, PC and PMMA were respectively dried at 120 °C for 10 h and 80 °C for 10 h, and GNs were dried at 50 °C for 10 h to reduce the moisture-induced thermal degradation during compounding. Then, three different blending sequences were employed to prepare PC/PMMA/GNs (70/30/0.05) composites: a one-step compounding sequence that all of the components were melt-blended in one processing, and two-step compounding sequence that pre-mixed PC/GNs or pre-mixed PMMA/GNs were first prepared and then were respectively blended with PMMA or PC in a second step. In the one-step compounding sequence, PC, PMMA and GNs were melt-blended together using a twin-screw co-rotating extruder (LHFD1-130718, Lab Tech Engineering Company Ltd.) with a temperature program of 240 °C, 245 °C, 250 °C, and 245 °C, and a screw speed of 200 rpm. In the two-step compounding sequence by two-step compounding sequence with pre-mixed PC/GNs, PC and GNs were first melt-blended together using an extruder, then the prepared PC/GNs pellets were melt-blended with PMMA using the extruder in a second step with the above-mentioned temperature program and screw speed. Similarly, in the two-step compounding sequence by two-step compounding sequence with pre-mixed PMMA/GNs, PMMA and GNs were first melt-blended together using an extruder, then the prepared PMMA/GNs pellets were melt-blended with PC using the extruder in a second step with the above-mentioned temperature program and screw speed. In comparison, PC/PMMA (70/30) blends pellets were also prepared using an extruder in one-step processing with the above-mentioned temperature program and screw speed. Then the blend and composites were all injection-molded (SZS-20, Wuhan Ruiming Experimental Instrument Co., Ltd., China) into standard dumbbell-shaped mechanical splines for morphological observation and characterization. The injection temperature and injection pressure were 250 °C and 0.51 MPa respectively; the closing time, first injection time and second injection time were 3 s, 3 s and 9 s respectively.

### 2.3 Characterization

A transmission electron microscope (JEM-200 CX, JEOL, Japan) was employed to study the distribution of GNs in the PC/PMMA blends and the phase morphology of the prepared samples at an accelerating voltage of 120 kV. Ultrathin sections with a thickness of 60–80 nm are cut from Izod bars perpendicular to the flow direction under cryogenic conditions using a LKB-5 microtome (LKB Co, Switzerland), and PC phase was dyed with RuO_4_.

A scanning electron microscope (FEI Instrument-QUANTU 250 FEG) was used to observe the phase morphology of the prepared samples at an accelerating voltage of 10 kV. The observed samples were placed in liquid nitrogen for 3 h and were brittle-fractured quickly; then, the PMMA dispersed phase on the fractured surface was etched with formic acid at 50 °C for 1 h and the sample surface was repeatedly washed 4–5 times with deionized water and then ultrasonicated in an ethanol solution for 10 min. Finally, the observed samples were vacuum-dried at 50 °C for 12 h and the etched surface of the specimens was gold sputtered for observation.

To quantitatively analyze the morphology of the fractured surface of the sample, the number-average domain diameters (*D*_n_) were obtained using the following equation:1
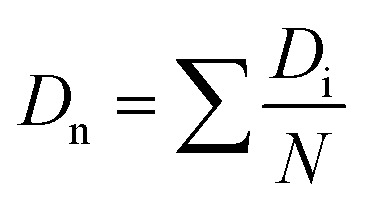
where *N* is the total number of dispersed domains and *D*_i_ is the particle diameter of a domain. More than 100 particles were analyzed for the accuracy of statistical samplings using the Nano Measurer software (Image-Pro, Media Cybernetics Inc.).

Contact angles between PC or PMMA or GNs and H_2_O were measured using a contact angle tester (DSA25, Germany Klux Company) at room temperature, and the contact angles between PC, PMMA, GNs and diiodomethane solution were measured under the same conditions. Based on the contact angle data obtained from the test, the surface tension of the polymers was calculated using Young's equation:

2 *γ*_SL_ + *γ*_LV_ cos *θ* = *γ*_SV_

where *γ*_SL_, *γ*_LV_, and *γ*_SV_ represent the interfacial energy of the solid/liquid, liquid/air, and solid/air interface respectively, and *θ* is the contact angle between the liquid and the solid surface.

Rheological properties of PC, PMMA, PC/GNs, PMMA/GNs and the composites prepared by different sequences were tested using a MARS rheometer (ARES, TA Instrument, New Castle, DE, USA) at 250 °C with parallel plates 25.4 mm in diameter. The frequency sweep range is 0.01–100 Hz, and the fixed strain is 1%.

The thermal conductivity of PC/PMMA blends and the PC/PMMA/GNs composites prepared by different sequences were tested using laser scattering thermal conductivity analyzer (LFA467, Geranys Natch Company). Preparation of the thermal conductivity test sample: the injection molded sample is pressed into a disc with a thickness of 1.2–1.3 mm and a diameter of 25.4 mm using a flat vulcanizer at 250 °C, and the test temperature is 25 °C.

Tensile tests of PC/PMMA blends and the composites prepared by different sequences were performed in a universal testing machine (CMT6104, Meters Industrial (China) Co., Ltd.) according to ISO 178 standard with a tensile rate of 5 mm min^−1^ and a clamp spacing of 58 mm. For each specimen, the data reported was the average of five specimens.

The samples were characterized by Raman spectroscopy (Renishaw). The Raman spectrometer uses 50 times lens, the excitation wavelength is 532 nm, the laser power is 1%, and the exposure time is 10 s.

The samples were characterized using an X-ray diffractometer (X'Pert PRO). The X-ray diffraction patterns were recorded using an X-ray power diffractometer with Cu Kα radiation (0.15406 nm) operating at a voltage of 45 kV and a current of 40 mA. Scans were performed over a 2*θ* range between 5° and 90° at a scan rate of 1° min^−1^.

## 3 Results and discussions

### 3.1 Migration and distribution of GNs in PC/PMMA blends

**Fig. 1:**
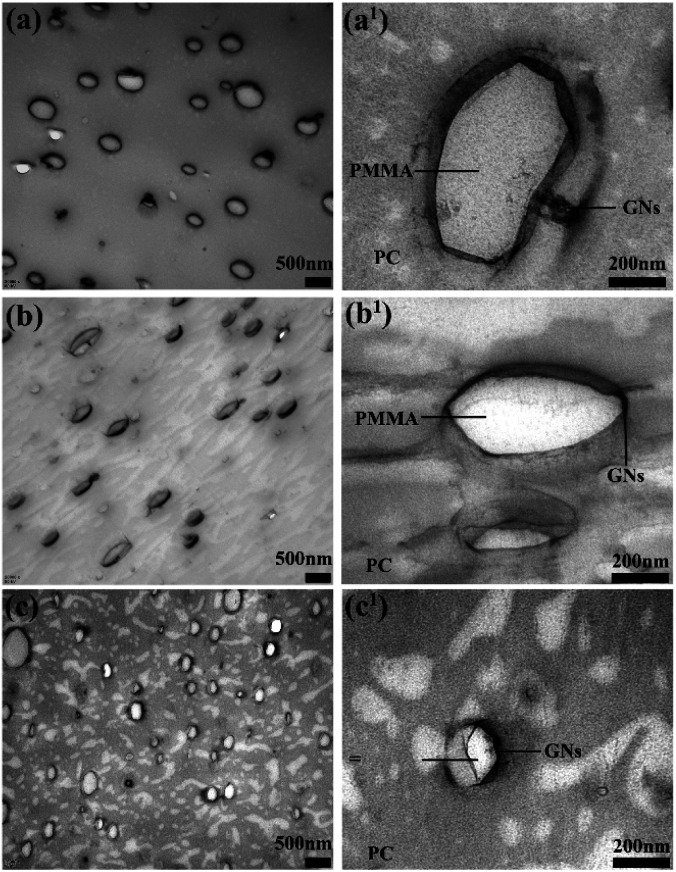
TEM micrographs of composites prepared by different melt blending sequences: (a) and (a^1^) PC/PMMA/GNs by two-step compounding with pre-mixed PMMA/GNs, (b) and (b^1^) PC/PMMA/GNs by two-step compounding with pre-mixed PC/GNs, (c) and (c^1^) PC/PMMA/GNs by one-step compounding.

Fig. 1 shows the TEM micrographs of PC/PMMA/GNs composites prepared by different blending sequences. As shown in Fig. 1, the PMMA dispersion phase and the PC matrix phase can be distinguished easily and PC and PMMA showed the typical sea-island morphology. From the TEM micrographs of composites prepared by two-step compounding with pre-mixed PMMA/GNs and PC/GNs (Fig. 1(a), (a1), (b) and (b1)), it is amazing to see that most GNs (dark line or platelets) localized in the interface of the PC/PMMA blends, signifying the migration of GNs from the PMMA phase to the interface or from the PC phase to the interface during melt blending. While for the composite prepared by one-step compounding (as seen in Fig. 1(c) and (c1)), majority of GNs (dark line or platelets) distributed in the interface of the PC/PMMA blends and slight GNs distributed in the PC phase, indicating that GNs also migrate during the melting process. Based on TEM observations, it is realized that the migration of GNs always existed in PC/PMMA/GNs composites during melt blending no matter how the GNs were introduced.

To study the migration mechanism of GNs, the wetting coefficient (*ω*_a_) was employed to predict the selective distribution of GNs in PC/PMMA blends. It can be calculated using eqn (3):^7^3
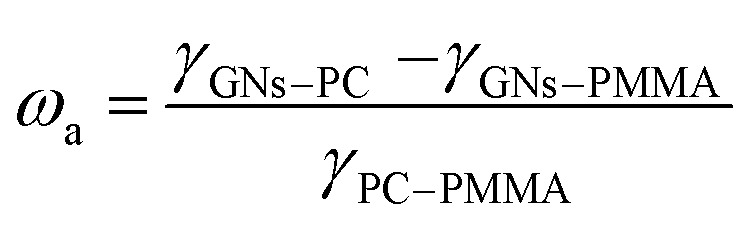
where *γ*_GNs–PC_, *γ*_GNs–PMMA,_ and *γ*_PC–PMMA_ are the interfacial tension between GNs and PC components, between GNs and PMMA components, and between PC and PMMA components. If *ω*_a_ > 1, GNs are preferentially localized in the PMMA phase. If *ω*_a_ < −1, GNs are preferentially localized in the PC phase. If −1 < *ω*_a_ < 1, GNs are localized in the interface of PC and PMMA.

The interfacial tensions among GNs and polymers were calculated using a harmonic-mean equation as the equation is more suitable to the interfacial energy between low-energy materials, such as polymers, organic liquids, and water,^13^ and the ultimate interfacial tensions calculated by surface tensions of the components (as shown in Table 1) are summarized in Table 2.^13–15^ Based on the interfacial tensions, *ω*_a_ was calculated according to eqn (3) and the result is −3.565 in this study, implying that GNs are preferentially localized in the phase of PC as *ω*_a_ < −1, demonstrating that the migration of GNs during melt blending was attributed to the thermodynamic reason of interfacial effects.

**Table 1:** Surface energy data of materials

Material	Surface tension at 25 °C (mJ m^−2^)			Temperature coefficient −d*γ*/d*T* (mJ m^−2^ °C)	Surface tension at 250 °C (mJ m^−2^)		
	*γ*	*γ* ^d^	*γ* ^p^		*γ*	*γ* ^d^	*γ* ^p^
PC	47.48	47.3	0.18	0.04^a^	38.48	38.33	0.15
PMMA	50.11	49.9	0.21	0.077^b^	32.79	32.65	0.14
GNs	48.07	46.21	1.86	0^c^	48.07	46.21	1.86

a Value for PC reported in ref. 14.

b Value for PMMA reported in ref. 13.

c Value for GNs reported in ref. 15.

**Table 2:** Interfacial tensions and wetting coefficients according to the harmonic mean equation

Material	Interfacial tension (mJ m^−2^)	Wetting coefficient, *ω*_a_	GNs location prediction
	Harmonic mean equation	Harmonic mean equation	
GNs–PC	2.189	−3.565	PC
GNs–PMMA	3.811		
PC–PMMA	0.455		

### 3.2 Effect of migration and selective distribution of GNs on the phase morphology of composites

**Fig. 2:**
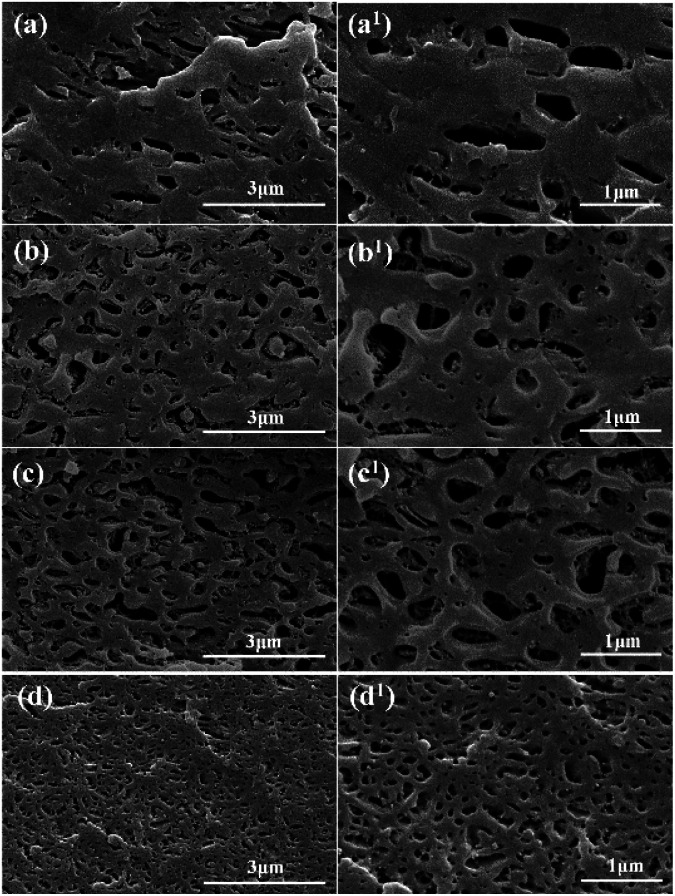
SEM images of composites etched by glacial formic acid. (a) and (a^1^) PC/PMMA blends; (b) and (b^1^) PC/PMMA/GNs by two-step compounding with pre-mixed PMMA/GNs; (c) and (c^1^) PC/PMMA/GNs by two-step compounding with pre-mixed PC/GNs; (d) and (d^1^) PC/PMMA/GNs by one-step compounding.

It is known that migration and selective distribution of layered nanoparticles showed great effects on the phase morphology of polymer composites. Fig. 2 exhibits the SEM micrographs and particle size distribution curve of PC/PMMA blends and PC/PMMA/GNs composites prepared by different sequences. As expected, all of the prepared samples showed the typical sea-island morphology (shown in Fig. 2(a)–(d)), where the hole domains indicate the PMMA dispersed phase extracted by formic acid.

As seen that the average particle size is quite different for different composites (see in Fig. 2(a1), (b1), (c1) and (d1)), it is calculated using the image analyzing software that the number-average diameter of dispersion phase was respectively 0.33 μm for PC/PMMA blends, 0.23 μm for the composite prepared by two-step compounding sequence with pre-mixed PMMA/GNs, 0.2 μm for the composite prepared by two-step compounding sequence with pre-mixed PC/GNs, and 0.12 μm for the composite prepared by one-step compounding sequence, indicating that the migration and selective distribution of GNs in the interface of PC and PMMA during the melting process showed great effects on the size of the dispersed phase.

**Fig. 3:**
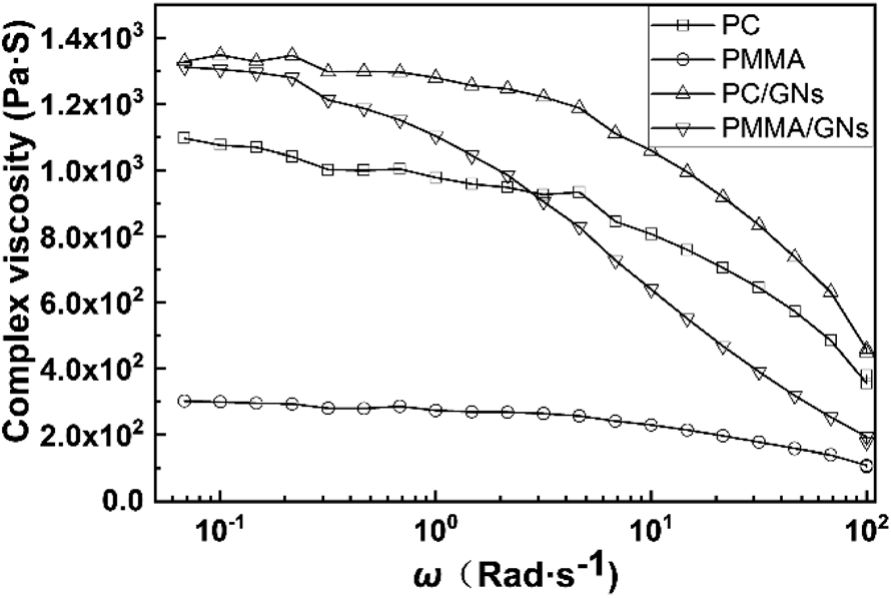
Logarithmic plot of complex viscosity (η*) of polymers and composites *versus* the angular frequency at 250 °C.

It is known from the TEM results that GNs always selectively distributed at the interface of PC and PMMA, which could play a role as compatibilizer to reduce the size of dispersed phase.^16,17^ Therefore, the dispersed phase size of all of the composites was smaller than that of the PC/PMMA blends. In addition, according to previous studies, the ultimate domain size of immiscible blends is determined by the balance between two opposing factors: droplet breakup and coalescence, which are greatly influenced by the phase separation of the polymer melt and the viscosity of matrix and dispersed phase.^18,19^ For the composite prepared by two-step compounding sequence with pre-mixed PMMA/GNs, although migration of GNs from the PMMA dispersed phase to the interface of PC and PMMA during melt blending could form a special “knife-like” effect to cut the dispersed phase melt apart to refine the dispersed phase size,^20^ the increased viscosity of PMMA with addition of GNs could balance the effect, while for the composite prepared by two-step compounding sequence with pre-mixed PC/GNs, because addition of GNs could greatly increase the melt viscosity of PC matrix phase (see in Fig. 3), preventing the coalescence of dispersed phase and reducing the dispersed phase size, the dispersed phase size of the composite prepared by two-step compounding sequence with pre-mixed PC/GNs was smaller than that of the composite prepared by two-step compounding sequence with pre-mixed PMMA/GNs. For the composite prepared by one-step compounding sequence, on the one hand, migration of GNs during the melt process could refine the dispersed phase, and, on the other hand, the GNs localized in the PC matrix phase could increase their melt viscosity to prevent the coalescence of the dispersed phase. The two reasons led to the smallest dispersed phase size of the composite prepared by one-step compounding sequence.

### 3.3 Mechanical properties of PC/PMMA/GNs composites

**Fig. 4:**
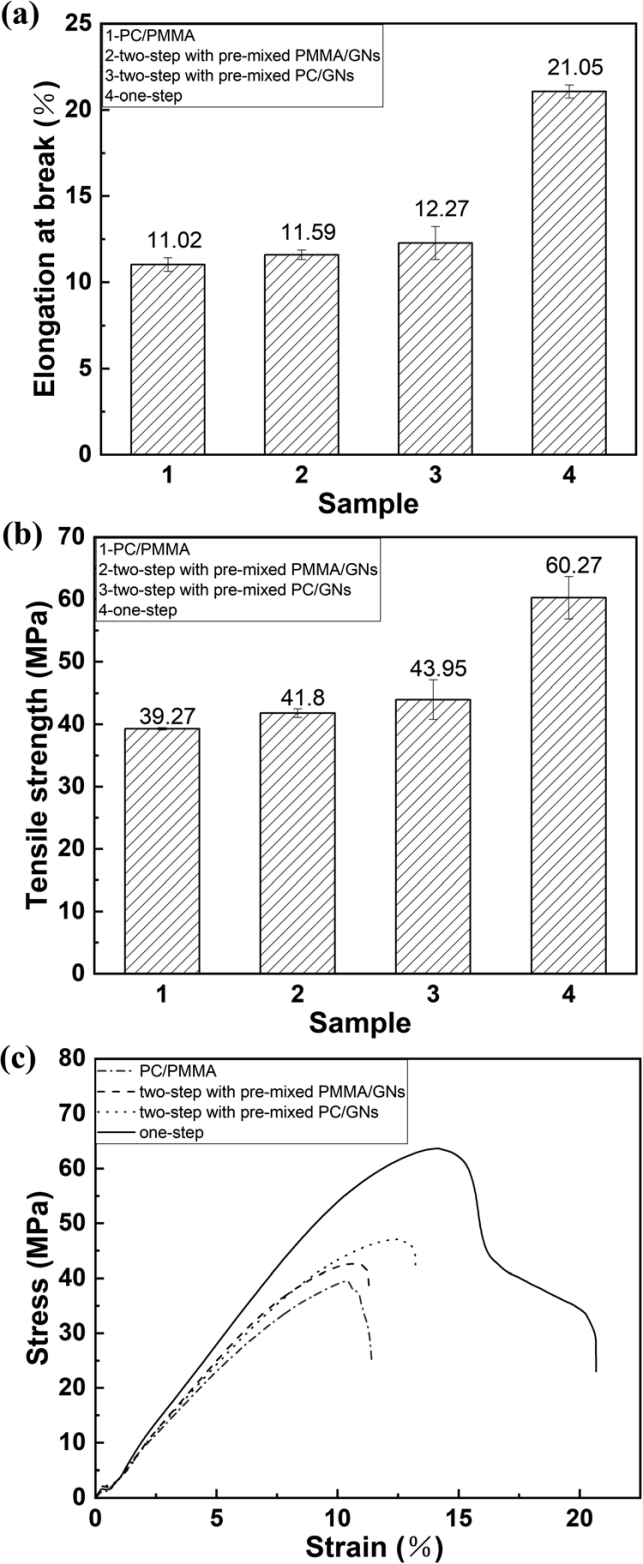
(a) Tensile strength, (b) elongation at break, (c) stress–strain curve of PC/PMMA blends and PC/PMMA/GNs composites prepared by different sequences.

Fig. 4 shows the tensile strength, elongation at break and stress–strain curve of the PC/PMMA blends and the PC/PMMA/GNs composites prepared by different blending sequences. Obviously, addition of GNs could increase the tensile strength and elongation of the blend, and the composite prepared by two-step compounding sequence with pre-mixed PC/GNs showed better tensile strength and elongation than that of the composite prepared by two-step compounding sequence with pre-mixed PMMA/GNs. It is noteworthy that the composite prepared by one-step compounding showed significantly enhanced strength and toughness, as shown in Fig. 4. Amazingly, the tensile strength and elongation of the composite were respectively increased by about 62.96% and 94.54% with only 0.05 wt% GNs addition, as compared to the PC/PMMA blends, signifying that the blending sequence of the composites and its effect on the migration and selective localization of nanoparticles showed great effects on the final properties of the composites.

**Fig. 5:**
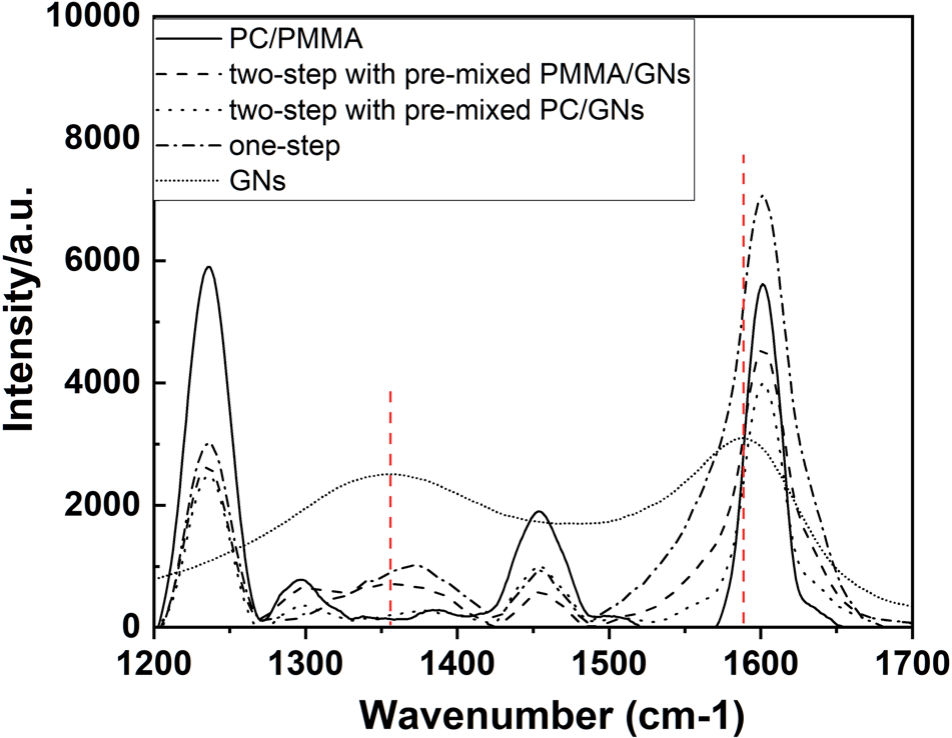
Raman spectrum of GNs, PC/PMMA blends and PC/PMMA/GNs composites prepared by different sequences.

It is known that the ultimate performance of nanoparticle/polymer ternary composites was determined by the dispersion of the nanoparticle and its interactions with polymers as well as the phase morphology and compatibility of the composites.^20^ To evaluate the interactions between GNs and polymer composites, the Raman test was carried out. Fig. 5 shows the Raman spectrum of GNs, PC/PMMA blends, and PC/PMMA/GNs composites, and the results are summarized in Table 3. Obviously, the D and G bands of neat GNs appeared at about 1355 cm^−1^ and 1588 cm^−1^, and the ratio of *I*_D_/*I*_G_ was about 0.51. However, in the composite systems, the D and G bands of GNs were shifted and the ratio of *I*_D_/*I*_G_ was decreased (as shown in Table 3), which means that there is a strong interaction between GNs and polymers,^21^ which was attributed to the improved mechanical property of the PC/PMMA/GNs composites relative to that of the PC/PMMA blends. Moreover, it is seen that the deviation degree of D and G bands in the composites prepared by two-step compounding sequence with pre-mixed PC/GNs and by one-step compounding sequence was relatively prominent (as shown in Table 3), implying that the interaction between GNs and polymers was stronger than that of composites prepared by two-step compounding sequence with pre-mixed PMMA/GNs. As a result, the tensile strength of the composites prepared by two-step compounding sequence with pre-mixed PC/GNs and by one-step compounding sequence was better than that of composites prepared by two-step compounding sequence with pre-mixed PMMA/GNs. Moreover, the strong interaction between GNs and polymers means that more GNs might rearrange and orient during the stretch process in the composites prepared by two-step compounding sequence with pre-mixed PC/GNs and by one-step compounding sequence due to the strong interaction,^22^ which could dissipate much energy and prevent crack growth. Therefore, the toughness of the composites prepared by two-step compounding sequence with pre-mixed PC/GNs and one-step compounding sequence was also better than that of composites prepared by two-step compounding sequence with pre-mixed PMMA/GNs.

**Table 3:** The corresponding D band, G band and *I*_D_/*I*_G_ value of the material in Raman spectrum

Material	D band, cm^−1^	G band, cm^−1^	*I*_D_/*I*_G_
GNs	1355.91	1588.02	0.51
PC/PMMA/GNs (two-step with pre-mixed PMMA/GN)	1358.69	1601.52	0.15
PC/PMMA/GNs (two-step with pre-mixed PC/GNs)	1376.79	1601.64	0.07
PC/PMMA/GNs (one-step)	1373.20	1601.33	0.14

**Fig. 6:**
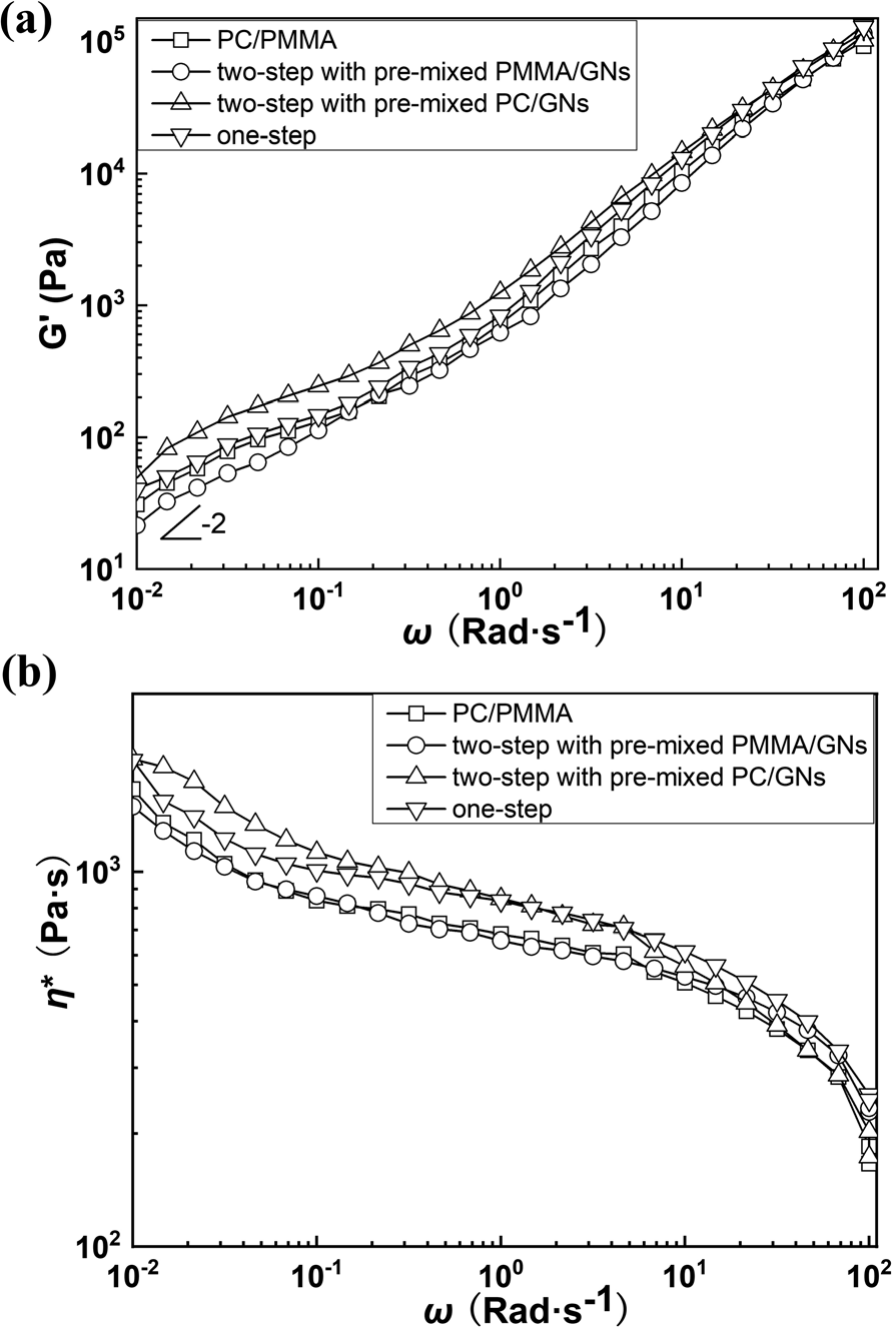
(a) Logarithmic plot of diagonal frequency of storage modulus (*G*′) of PC/PMMA blends and PC/PMMA/GNs composites prepared by different sequences at 250 °C. (b) Logarithmic plot of complex viscosity (η*) and diagonal frequency of PC/PMMA blends and PC/PMMA/GNs composites prepared by different sequences.

**Fig. 7:**
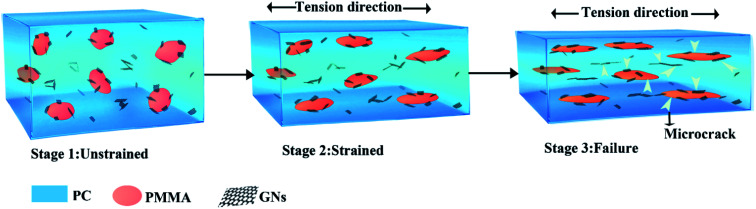
Schematic of the strengthening and toughening mechanism of the PC/PMMA/GNs composite prepared by one-step compounding.

However, it is known from the SEM and TEM results that GNs are always selectively distributed on the interface of PC and PMMA during the melting process, which could enhance the compatibility of the matrix and dispersed phases, making the PMMA dispersed phase deform along the direction of external tensile stress during stretching to dissipate energy.^17^ Furthermore, it is seen that the dispersed phase size of composites prepared by two-step compounding sequence with pre-mixed PC/GNs and one-step compounding sequence was relatively small; this small dispersed phase implied that there were more stress concentration points to dissipate energy in the system,^23,24^ which was another explanation for the relatively better mechanical property of composites prepared by two-step compounding sequence with pre-mixed PC/GNs and one-step compounding sequence. However, the unexpected enhanced tensile strength and elongation of the composite prepared by one-step compounding suggested that some other factors also affected the mechanical properties of the composite. Therefore, the rheological test was employed to characterize the structure of the composites. Fig. 6 exhibits the storage modulus of the PC/PMMA blends and PC/PMMA/GNs composites prepared by different sequences *versus* frequency at 250 °C. As shown from Fig. 6(a) that the terminal rheological behavior of the low-frequency region of PC/PMMA/GNs composites prepared by one-step compounding was slightly different from that of other composites, and the slope value was deviated from the linear viscoelastic theory, suggesting that some GNs network structures were formed in the composite. In addition, the relatively larger shear dilution index calculated from the curve of lg η* *versus* lg *ω* indicated that dispersion of GNs in the composite was also better than that of others (as shown in Fig. 6(b)).^25^ The well dispersion and certain degree network structure could greatly enhance the tensile strength of the composite prepared by one-step compounding. Meanwhile, good dispersion of GNs implied that there were more stress concentration points to dissipate energy and prevent crack growth, which could also significantly enhance the toughness of the composite. In conclusion, the relative strong interaction between GNs and polymers and the small dispersed phase size as well as the certain degree network structure and the better dispersion of GNs led to the unexpectedly reinforced and toughened property of the composite prepared by one-step compounding. Based on the above-mentioned analysis, strengthening and toughening mechanism were revealed and illustrated by the schematic drawing of Fig. 7.

### 3.4 Thermal conductivity of PC/PMMA/GNs composites

**Fig. 8:**
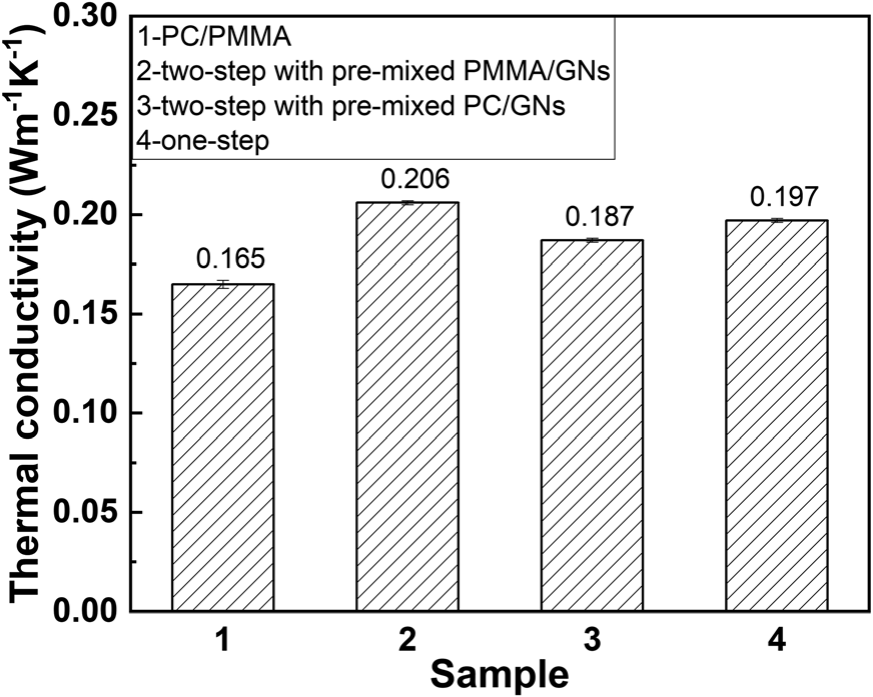
Thermal conductivity of PC/PMMA blends and PC/PMMA/GNs composites prepared by different sequences.

**Fig. 9:**
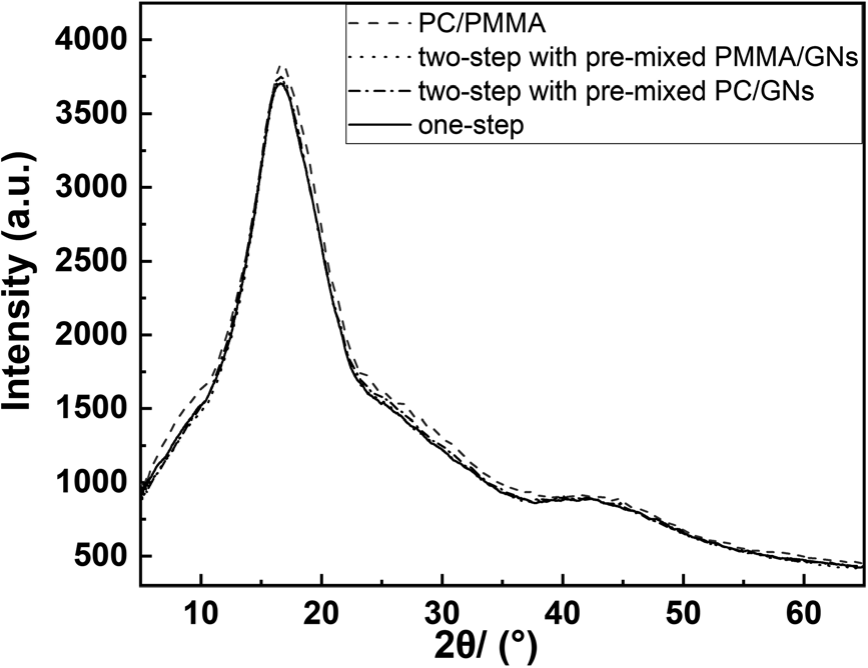
WAXD spectra of composite materials.

Fig. 8 shows the thermal conductivity of PC/PMMA/GNs composites prepared by different sequences. As expected, the addition of GNs could increase the thermal conductivity of the composites. However, unexpectedly, the composite prepared by two-step compounding sequence with pre-mixed PMMA/GNs showed the best thermal conductivity followed by the composite prepared by one-step compounding, which was quite different from the results of mechanical property. It is seen that the thermal conductivity of the composite prepared by two-step compounding sequence with pre-mixed PMMA/GNs and one-step compounding was respectively increased by about 23.5% and 19.4% compared to that of the PC/PMMA blends with the addition of only 0.05 wt% GNs. According to previous studies,^5,26,27^ the thermal conductivity of nanoparticle/polymer composites was mainly determined by the crystalline degree of the composite and heat conduction channel constructed by the nanoparticles. To gain an in-depth understanding of the results of thermal conductivity, WAXD was employed to study the crystalline degree of the composite. As shown in Fig. 9, the crystalline degree of the composites was similar, signifying that its thermal conductivity primarily depends on the heat conduction network structure constructed by GNs.

According to TEM micrographs, irrespective of the blending sequence, some GNs will be distributed on the interface between PC and PMMA. According to the value of *ω*_a_, for the pre-blended components of GNs and PMMA, GNs will migrate from PMMA to PC, and some GNs will be distributed on the interface between PC and PMMA during migration. Therefore, the composite prepared by pre-blending GNs and PMMA has more GNs distributed on its interface, forming a more perfect heat conduction path, and its heat conduction performance is also the best. However, in the composite prepared by one-step compounding, only part of GNs were distributed on the interface of PC and PMMA to form the heat conduction channel, as GNs were preferentially localized in the PC phase. Similarly, it is deduced that only a few GNs localized on the interface of PC and PMMA to form the heat conduction channel in the composite prepared by two-step compounding sequence with pre-mixed PC/GNs. Therefore, it is accepted that the composite prepared by two-step compounding sequence with pre-mixed PMMA/GNs showed the best thermal conductivity, and the composite prepared by one-step compounding showed relatively better comprehensive performance.

## 4 Conclusion

In this study, three different blending sequences were employed to tune the migration and selective localization of GNs, and the effects of the migration and selective localization of GNs on the structure and performance of PC/PMMA blends were studied. The TEM results indicated that the migration of GNs always existed in PC/PMMA blends during melt blending no matter how the GNs were introduced, and part of GNs were exclusively distributed at the interface of PC and PMMA phases due to interfacial effects. The mechanical property study indicated that the migration and selective localization of GNs could greatly improve the comprehensive mechanical properties of the composites, and the composite prepared by one-step compounding showed significantly enhanced strength and toughness with the addition of only 0.05 wt% GNs. In comparison to the PC/PMMA blends, the tensile strength and elongation of the composite were respectively increased by about 62.96% and 94.54%. It was demonstrated that the reinforcing and toughening mechanism were attributed to the relative strong interaction between GNs and polymers, and the relatively small dispersed phase size and interfacial compatibilizing effect caused by the migration and selective localization of GNs as well as the well dispersion and certain degree network structure of GNs in PC matrix phase. The schematic of strengthened and toughened mechanism can be inferred from the above analysis. Furthermore, the composite prepared by one-step compounding also showed improved thermal conductivity at the same time. It is believed that tuning the migration and selective localization of GNs opens up new avenues for the development of high-performance polymer composites.

## Conflicts of interest

There are no conflicts to declare.

## Supplementary Material

## References

[cit1] Wang X., Gao Y., Li X., Xu Y., Jiang J., Hou J., Li Q., Turng L. (2017). Selective localization of graphene oxide in electrospun polylactic acid/poly(ε-caprolactone) blended nanofibers. Polym. Test..

[cit2] Liu T., Zhang H., Zuo M., Zhang W., Zhu W., Zheng Q. (2019). Selective location and migration of poly(methyl methacrylate)-grafted clay nanosheets with low grafting density in poly(methyl methacrylate)/poly(styrene-co-acrylonitrile) blends. Compos. Sci. Technol..

[cit3] Botlhoko O., Ray S., Ramontja J. (2018). Influence of functionalized exfoliated reduced graphene oxide nanoparticle localization on mechanical, thermal and electronic properties of nanobiocomposites. Eur. Polym. J..

[cit4] Bai L., Sharma R., Cheng X., Macosko C. (2018). Kinetic Control of graphene localization in co-continuous polymer blends via melt compounding. Langmuir.

[cit5] Huang J., Zhu Y., Xu L., Chen J., Jiang W., Nie X. (2016). Massive enhancement in the thermal conductivity of polymer composites by trapping graphene at the interface of a polymer blend. Compos. Sci. Technol..

[cit6] Xie G., Yu J., Qin S., Sun J., Yang Z., Wei L., Ji Y., He W., Xu G. (2018). Selective localization of organic montmorillonite nanoparticles in multilayered high-density polyethylene/polyamide 6 composites. Adv. Polym. Technol..

[cit7] Li L., Ruan W., Zhang M., Rong M. (2017). Studies on the selective localization of multi-walled carbon nanotubes in blends of poly(vinylidene fluoride) and polycaprolactone. eXPRESS Polym. Lett..

[cit8] Pang Y., Yang J., Curtis T., Lou S., Huang D., Feng Z., Morales-Ferreiro J. (2019). *et al.*, Exfoliated graphene leads to exceptional mechanical properties of polymer composite films. ACS Nano..

[cit9] Mahmoud M., Maher E. F., Safwat A., Richard B. K., Peter M., Jun M. (2018). Compact, flexible conducting polymer/graphene nanocomposites for supercapacitors of high volumetric energy density. Compos. Sci. Technol..

[cit10] Yang W., Gong Y., Zhao X., Liu T., Zhang Y., Chen F., Fu Q. (2019). Strong and highly conductive graphene composite film based on the nanocellulose-sssisted dispersion of expanded graphite and incorporation of poly(ethylene oxide). ACS Sustainable Chem. Eng..

[cit11] Amani M., Sharif M., Kashkooli A., Rahnama N., Fazli A. (2015). Effect of mixing conditions on the selective localization of graphite oxide and the properties of polyethylene/high-impact polystyrene/graphite oxide nanocomposite blends. RSC Adv..

[cit12] Tu C., Nagata K., Yan S. (2017). Key factor of graphene localization on electrical conductive properties of graphene filled polyethylene/polypropylene composites during melt blending. J. Mater. Sci. Res..

[cit13] Wu S. (1973). Polar and nonpolar interactions in adhesion. J. Adhes..

[cit14] Mehrabi R., Ghasemi I., Karrabi M., Azizi H. (2013). Nanocomposites based on polycarbonate/poly (butylene terephthalate) blends effects of distribution and type of nanoclay on morphological behaviour. J. Vinyl Addit. Technol..

[cit15] Shen Y., Zhang T., Yang J., Zhang N., Huang T., Wang Y. (2017). Selective localization of reduced graphene oxides at the interface of PLA/EVA blend and its resultant electrical resistivity. Polym. Compos..

[cit16] Tiwari S., Hatui G., Oraon R., Adhikari A., Nayak G. (2017). Mixing sequence driven controlled dispersion of graphene oxide in PC/PMMA blend nanocomposite and its effect on thermo-mechanical properties. Curr. Appl. Phys..

[cit17] Yan W., Qin S., Guo J., Zhang M., He M., Yu J. (2012). Morphology and mechanical properties of acrylonitrile-butadiene-styrene (ABS)/polyamide 6 (PA6) nanocomposites prepared via melt mixing. J. Macromol. Sci., Part B: Phys..

[cit18] Jmh J., Heh M. (1993). Droplet breakup mechanisms: stepwise equilibrium versus transient dispersion. J. Rheol..

[cit19] Mallick S., Kar P., Khatua B. (2012). Morphology and properties of nylon 6 and high density polyethylene blends in presence of nanoclay and PE-g-MA. J. Appl. Polym. Sci..

[cit20] Zhu Y., Ma H., Tong L., Fang Z. (2008). “Cutting effect” of organoclay platelets in compatibilizing immiscible polypropylene/polystyrene blends. J. Zhejiang Univ., Sci., A.

[cit21] Sain P., Goyal R., Prasad Y., Jyoti, Sharma K., Bhargava A. (2015). Few-layer-graphene/polycarbonate nanocomposites as dielectric and conducting material. J. Appl. Polym. Sci..

[cit22] Wang M., Ruan W., Huang Y., Ye L., Rong M., Zhang M. (2012). A strategy for significant improvement of strength of semi-crystalline polymers with the aid of nanoparticles. J. Mater. Chem..

[cit23] Wu S. (1988). A generalized criterion for rubber toughening: the critical matrix ligament thickness. J. Appl. Polym. Sci..

[cit24] Wu S. (1985). Phase structure and adhesion in polymer blends: a criterion for rubber toughening. Polymer.

[cit25] Samyn F., Bourbigot S., Jama C., Bellayer S., Nazare S., Hull R., Fina A., Castrovinci A., Camino G. (2008). Characterisation of the dispersion in polymer flame retarded nanocomposites. Eur. Polym. J..

[cit26] Kim J., Kim G. (2014). Effect of orientation and content of carbon based fillers on thermal conductivity of ethylene-propylene-diene/filler composites. J. Appl. Polym. Sci..

[cit27] Ge T., Shu W. (2018). Effect of different nucleating agents on crystallization properties of thermal conductivity PBT. Plast. Sci. Technol..

